# Clinical results of Hi-tech Knee II total knee arthroplasty in patients with rheumatoid athritis: 5- to 12-year follow-up

**DOI:** 10.1186/1749-799X-7-9

**Published:** 2012-02-22

**Authors:** Hajime Yamanaka, Ken-ichiro Goto, Munetaka Suzuki

**Affiliations:** 1 Department of Orthopaedic Surgery, National Hospital Organization Shimoshizu Hospital, 934-5 Shikawatashi, Yotsukaido City, Chiba 284-0003, Japan

## Abstract

**Background:**

Total knee arthroplasty (TKA) is a common form of treatment to relieve pain and improve function in cases of rheumatoid arthritis (RA). Good clinical outcomes have been reported with a variety of TKA prostheses. The cementless Hi-Tech Knee II cruciate-retaining (CR)-type prosthesis, which has 6 fins at the anterior of the femoral component, posterior cruciate ligament (PCL) retention, flat-on-flat surface component geometry, all-polyethylene patella, strong initial fixation by the center screw of the tibial base plate, 10 layers of titanium alloy fiber mesh, and direct compression molded ultra high molecular weight polyethylene (UHMWPE), is appropriate for TKA in the Japanese knee.

The present study was performed to evaluate the clinical results of primary TKA in RA using the cementless Hi-Tech Knee II CR-type prosthesis.

**Materials and methods:**

We performed 32 consecutive primary TKAs using cementless Hi-Tech Knee II CR-type prosthesis in 31 RA patients. The average follow-up period was 8 years 3 months. Clinical evaluations were performed according to the American Knee Society (KS) system, knee score, function score, radiographic evaluation, and complications.

**Results:**

The mean postoperative maximum flexion angle was 115.6°, and the KS knee score and function score improved to 88 and 70 after surgery, respectively. Complications, such as infection, occurred in 1 patient and revision surgery was performed. There were no cases of loosening in this cohort, and prosthesis survival rate was 96.9% at 12 years postoperatively.

**Conclusion:**

These results suggest that TKA using the cementless Hi-Tech Knee II CR-type prosthesis is a very effective form of treatment in RA patients at 5 to 12 years postoperatively. Further long-term follow-up studies are required to determine the ultimate utility of this type of prosthesis.

## Background

The affected joints in patients with rheumatoid arthritis (RA) show chronic proliferative synovitis, which has been implicated in the destruction of articular cartilage and bone, resulting in joint disability [1]. Good clinical results of total knee arthroplasty (TKA) in patients with RA have been reported using several types of prosthesis [2-5]. The decisions regarding whether the posterior cruciate ligament (PCL) should be replaced or retained and whether the prosthesis should be fixed using cement or not are made at the surgeon's discretion. Long-term (≥ 10 years) follow-up studies of cruciate-retaining (CR) TKA without cement fixation of the prosthesis in patients with RA have indicated prosthesis survival rates of over 90% [6-8].

**Figure 1:**
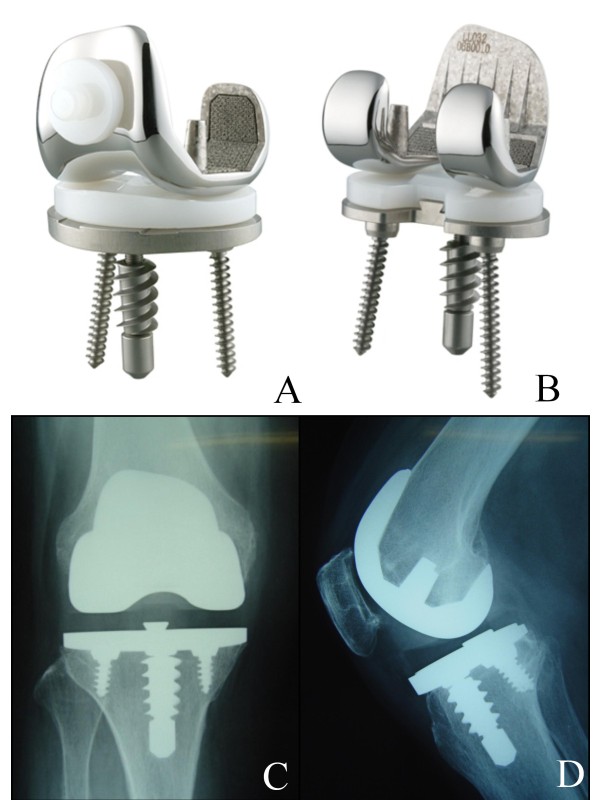
**A: Anterior view of the Hi-tech knee II prosthesis CR type component. Note the polyethylene geometry with flat surface**. **B**: Posterior view of the Hi-tech knee II prosthesis CR type component. Femoral component demonstrating the porous titanium coating and have 6 fins at anterior portion. **C**: Anteroposterior radiograph of Hi-tech knee II prosthesis CR type. **D**: Lateral radiograph of Hi-tech knee II prosthesis CR type.

Hi-Tech Knee II CR-type cementless TKA (Nakashima Medical, Okayama, Japan) was developed in 1994 at Chiba University and is currently in clinical use, having been applied in more than 1600 cases to date (Figure 1A-D). This prosthesis is an appropriate design for the Japanese knee, with 6 fins at the anterior of the femoral component, PCL retention, flat-on-flat surface component geometry, all-polyethylene patella fixed without cement, strong initial fixation by the center screw of the tibial base plate, 10 layers of titanium alloy fiber mesh, and direct compression molded ultra high molecular weight polyethylene (UHMWPE).

The present study was performed to evaluate the mid-term (5-12 year) results of Hi-Tech Knee II CR-type cementless TKA in patients with RA. This is the first report of the mid-term results regarding the use of this prosthesis.

All patients gave their informed consent prior inclusion in the study.

## Materials and methods

### Patients

Thirty-two Hi-Tech Knee II CR-type cementless primary TKAs were performed at our institution in 31 RA patients between April 1998 and August 2005. The study population consisted of 30 women and 1 man with a mean age of 64.4 years (range: 39-78 years) at surgery. The mean body mass index of the 31 patients was 25.5 (range: 20.5-32.3). The average follow-up period was 8 years 3 months (range: 5 year 3 months-12 years 3 months).

All patients fulfilled the diagnostic criteria of the American College of Rheumatology (ACR) [9], and had Larsen grade IV or V radiographic involvement at surgery [10]. The mean duration was 13.5 years (range: 5-32 years). Twenty-three patients (74%) were receiving active oral steroid therapy, and the average steroid dose was 4.8 mg (range: 1-15 mg) daily.

This prosthesis was not indicated for use in cases with revision surgery, mutilating type RA, absent or attenuated PCL was observed in operation, severe deformity (lower limb AP varus or valgus alignment > 30°), severe knee stiffness (range of knee movement arc < 50°), or severe instability of the knee (varus or valgus instability > 30°, posterior sagging instability). We did not consider metaphyseal defects of the tibia as a contraindication for use of this prosthesis.

### Surgical technique

A midline, longitudinal skin incision was used and the joint was opened through the median parapatellar approach. The PCL was preserved in all cases. Synovium existing to the suprapatellar pouch, around the cruciate ligaments, bilateral gutter, and posterior capsule were resected as much as possible. Bone cuts were made using an intramedullary guide on both the femur and tibia oriented by the measured cut technique. Cancellous bone chips were used to fill any bony defects and the tibial component was fixed with three screws to ensure initial stability. All patellae were also fixed with all-polyethylene components without cement. All procedures were performed with use of a tourniquet. Postoperative antibiotic (cefazolin sodium) was administered four times in 2 days. All patients received the same deep vein thrombosis prophylaxis using 100 mg of acetylsalicylic acid for 7 days.

### Postoperative care

Range of motion exercise and full weight-bearing transfer to a wheelchair were begun on the second postoperative day, and full weight-bearing walking exercise was started on the fifth day. Patients were discharged when they could walk with a T-cane and tolerate stair climbing.

### Methods and evaluation

Patients were evaluated preoperatively and postoperatively by one of two senior joint replacement surgeons (HY, KG) independent of the surgeon performing the operation at 4 weeks, 6 months, and 1 year, and then every 1 year thereafter.

**Figure 2:**
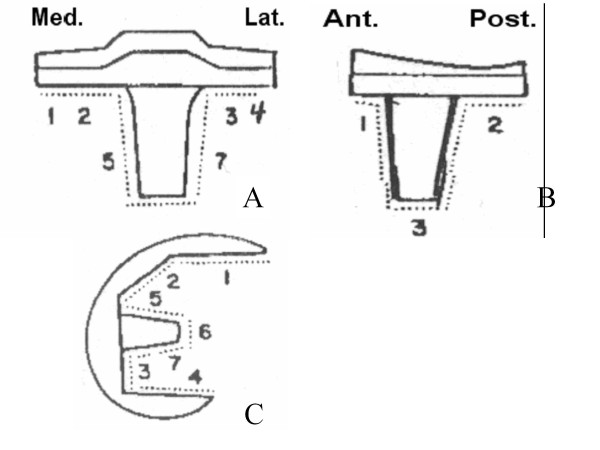
**The Knee Society Roentgenographjc Evaluation System**. **A**: Anteroposterior view of representative tibial component. **B**: Lateral view of representative tibial component. **C**: Lateral view of representative femoral component.

Clinical evaluations were performed according to the Knee Society (KS) system [11], which separately assesses the mechanical and functional aspects of the knee joint. Final follow-up standard anteroposterior and lateral radiographs were evaluated for loosening, radiolucent line, and subsidence according to the method of the Knee Society roentgenographic evaluation and score system [12]. The radiographs were taken according to the standard protocol described previously [13]. Weight-bearing anteroposterior (AP), lateral, and skyline image radiographs were taken before and after the operation. The femorotibial angle (FTA) of the knee was evaluated on AP weight-bearing radiographs. Radiolucencies were measured according to the zone described by the Knee Society (Figure 2A-C). AP and mediolateral instability were evaluated at each visit.

Complications, including infection, fracture, *etc*., were also assessed. Statistical analysis was performed using the paired *t *test for comparison of preoperative and postoperative Knee Society scores.

We calculated prosthesis survival according to the Kaplan-Meier method using revision for any cause, revision attributable to loosening, or infection [14].

## Results

### Clinical results

The average Knee Society knee rating score improved from 32 (range: 20-65) preoperatively to 88 (range: 44-96) at final follow-up evaluation. The average Knee Society function score was 41 (range: 25-93) preoperatively, and also improved to 70 (range: 50-95) at final follow-up. None of the patients showed posterior translation of the tibia of greater than 10 mm in the posterior drawer test. Twenty-six knees (79%) were pain-free and 7 knees (21%) had mild or occasional pain postoperatively.

Ten patients could walk for 30 to 60 min, 17 for 30 to 60 min, and 6 for less than 10 min. Eleven patients required no walking aid, while 17 patients used walking aids outdoors, and 5 patients required them permanently. The mean preoperative maximum flexion angle was 105.4°, and that postoperatively at the latest follow-up was 115.6° (*P *< 0.05). Maximum extension angles were -12.5° and -3.3°, respectively (*P *< 0.005). Good valgus-varus stability was maintained throughout the follow-up period.

### Radiographic results

All 32 patients in whom radiographic follow-up was performed were assessed for radiolucency at the bone-implant interface. Fourteen radiolucencies were identified (44%): 5 were seen at the femoral component (zone 1: 3 knees; zone 4: 1 knee; zone 5: 1 knee) and 9 were at the tibial component (AP view, zone 1: 5 knees; zone 3: 4 knees; lateral view, none). All measured less than 1 mm distance and were non-progressive. No radiolucent line was seen in the patella component, and there was no evidence of radiographic loosening at follow-up. Subsidences were detected in 7 knees (22%), but none were progressive. The subsidence may have been due to incorrect cutting of the tibial bone or poor bone strength.

The average postoperative FTA alignment was 174.7° (range: 170°-178°), and we found no differences in alignment throughout the follow-up period.

### Complications

Complications occurred in two knees (6.1%): early deep infection in one knee and supracondylar fracture of the femur in the other. The case of infection required revision surgery, while the case of fracture was healed by conservative treatment.

There were no cases of late deep infection, nerve palsy, pulmonary embolism, deep vein thrombosis, or patella fracture.

Prosthetic survival rate was 96.9% at 12 years postoperatively (95% confidence interval [CI], 86.6%-100%).

## Discussion

TKA is a reliable form of treatment to relieve pain and improve function in patients with RA. Factors that influence prosthetic survival include the design of the component, the method of fixation (cemented or cementless), and the preservation or excision of the PCL [15-18]. It remains unclear whether it is best to retain or excise the PCL, and whether cement should or should not be used for implant fixation.

There have been several reports describing good long-term results of cementless TKA for RA, with prosthetic survival rate of over 90% with follow-up periods of more than 10 years [2-5]. However, there have been few reports regarding the long-term outcome of cementless CR-type TKA [6-8]. The results of the present study suggested that the clinical results of Hi-Tech Knee II CR-type cementless TKA in RA patients are satisfactory.

Cement has disadvantages related to toxicity [19], reduced bone stock at revision, and difficulty in treating infections [20]. However, early fixation after cementless TKA is the main problem related to stability of the prosthesis compared with cemented fixation [21]. The Hi-Tech Knee II CR-type component is coated with fiber mesh consisting of ten layers of titanium, designed to promote mechanical fixation by bone ingrowth [22]. The radiolucent line indicates the results of incorrect cutting of the femur and tibia. The width of this line is not progressive, and none of the patients in the present study had loosening, suggesting that adequate fixation can be achieved due to bony ingrowth.

The choice of whether to use a PCL-retaining design or posterior-stabilized design for TKA is based on limited data. It has been reported that there are no difference in clinical outcome between PCL retention and PCL removal, and conversely that the PS design results in a better range of motion and easier operation technique. Conditt [23] reported that substitution of the PCL with a spine and cam mechanism may not fully restore the functional capacity of the PCL, particularly in high-demand activities that involve deep flexion, squatting, kneeling, and gardening.

PCL substitution is believed to prevent posterior subluxation of the tibia in addition to enhancing roll-back during deep knee flexion. It has been suggested that PCL substitution allows greater conformity, which in turn results in less stress in the polyethylene tibial base plate. In this study, there were no cases of subluxation or loosening of the tibial base plate. Shai [24] reported the prosthesis survival rate of 97% at 13 years in the PCL-retaining procedure, and Rodriguez [25] also reported the rate of 91% at 15 years.

The disease state of the RA knee at the time of arthroplasty dictates whether the PCL is retained or sacrificed and whether cement is or is not used. Therefore, we did not use this prosthesis for all RA knees. The contraindications for use of this prosthesis include cases with mutilating type RA, severe deformity, severe knee stiffness, and severe instability of the knee. We did not consider metaphyseal tibial defect as a contraindication for use of this prosthesis.

There were some limitations in this study. First, this study was performed in a select patient population, and selection bias may have influenced the clinical outcomes. Second, the number of patients was small, which may have led to small-sample bias. Third, we did not perform blinded radiographic analysis. Fourth, the follow-up period was relatively short. Finally, this was a retrospective study.

In this study, there were no indications of problems associated with retention of the PCL and cementless fixation. However, attention should be paid to cases with large bony defects of the knee and with mutilating types of joint destruction. Appropriate selection of patient, prosthesis, and operative technique may lead to good clinical results, even using a CR-type prosthesis and cementless fixation.

## Conclusions

Although some concerns still remain, the satisfactory clinical and radiological results of the present study 5-12 years postoperatively support the use of this prosthesis in cases of RA. However, the results did not suggest that this prosthesis is superior to other cemented and PS type prostheses for RA patients. The ultimate utility of this type of prosthesis should be judged based only on its long-term clinical outcome, and further studies are therefore required.

## Competing interests

The authors declare that they have no competing interests.

## Authors' contributions

KG and MS made substantial contributions to study conception and design. All authors approved the final version of this article.

## References

[B1] Scott DL Grindulis KA Struthers GR Coulton BL Popert AL Bacon PA Progression of radiological changes in rheumatoid arthritis Ann Rheum Dis 1984 43 1 8 17 10.1136/ard.43.1.8 6696524 PMC1001208

[B2] Knutso K Tjornstrand B Lindgren L Survival of knee arthroplasties for rheumatoid arthritis Acta Orthop Scand 1985 56 5 422 425 10.3109/17453678508994363 4072664

[B3] Laskin RS O'Flynn HM Total knee replacement with posterior cruciate ligament retention in rheumatoid arthritis: problem and complications Clin Orthop Relat Res 1997 345 24 28 9418617

[B4] Wolfe F Zwillich SH The long-term outcomes of rheumatoid arthritis: a 23-year prospective, longitudinal study of total joint replacement and its predictors in 1600 patients with rheumatoid arthritis Arthritis Rheum 1998 41 6 1072 1082 10.1002/1529-0131(199806)41:6<1072::AID-ART14>3.0.CO;2-G 9627017

[B5] Ito J Koshino T Okamoto R Saito T 15-year follow-up study of total knee arthroplasty in patients with rheumatoid arthritis J Arthoplasty 2003 18 8 984 992 10.1016/S0883-5403(03)00262-6 14658102

[B6] Gill GS Joshi AB Long-term results of retention of the posterior cruciate ligament in total knee replacement in rheumatoid arthritis J Bone Joint Surg (Br) 2001 83 4 510 512 10.1302/0301-620X.83B4.11398 11380120

[B7] Meding JB Keating EM Ritter MA Faris PM Berend ME Long-term followup of posterior-cruciate-retaining TKR in patients with rheumatoid arthritis Clin Orthop Relat Res 2004 428 146 152 15534535 10.1097/01.blo.0000147134.52561.64

[B8] Sharm S Nicol F Hullin MG McCreath SW Long-term results of the uncemented Low Contact Stress total knee replacement in patients with rheumatoid arthritis J Bone Joint Surg (Br) 2005 87 8 1077 1080 10.1302/0301-620X.87B8.16133 16049242

[B9] Arnett FC Edworthy SM Bloch DA McShane DJ Fries JF Cooper NS Healey LA Kaplan SR Liang MH Luthra HS Thomas A Medsger J Mitchell DM Neustadt DH Pinals RS Schaller JG Sharp JT Wilder RL Hunder GG The American rheumatism association 1987 revised criteria for the classification of rheumatoid arthritis Arthritis Rheum 1988 31 3 315 324 10.1002/art.1780310302 3358796

[B10] Larsen A Dale K Eek M Radiographic evaluation of rheumatoid arthritis and related conditions by standard reference films Acta Radiol Diagn 1977 18 4 481 491 10.1177/028418517701800415 920239

[B11] Insall JN Dorr LD Scott RD Scott WN Rationale of the knee society clinical rating system Clin Orthop Relat Res 1989 248 13 14 2805470

[B12] Ewald FC The Knee Society total knee arthroplasty roentgenographic evaluation and scoring system Clin Orthop Relat Res 1989 248 9 12 2805502

[B13] Rand JA Cement or cementless fixation in total knee arthroplasty? Clin Orthop Relat Res 1991 273 52 62 1959287

[B14] Kaplan E Meier P Nonparametric estimation from incomplete observations J Am Stat Assoc 1958 53 282 457 481 10.2307/2281868

[B15] Akizuki S Takizawa T Horiuchi H Fixation of a hydroxyapatite-tricalcium phosphate-coated cementless knee prosthesis L Bone J Surg (Br) 2003 85 8 1123 1127 10.1302/0301-620X.85B8.13836 14653592

[B16] Khaw FM Kirk LMG Morris RW Gregg PJ A randomized, controlled trial of cemented versus cementless press-fit condylar total knee replacement J Bone J Surg (Br) 2002 84 5 658 666 10.1302/0301-620X.84B5.12692 12188480

[B17] Hirsh HS Lotke PA Morrison LD The posterior cruciate ligament in total knee surgery: save, sacrifice or substitute? Clin Orthop Relat Res 1994 309 63 68 7994978

[B18] Clark CR Rorabeck CH MacDonald S Posterior-stabilized and cruciate-retaining total knee replacement: a randomized study Clin Orthop Relat Res 2001 392 209 212 10.1097/00003086-200111000-00025 11716384

[B19] Jones LC Hungerford DS Cement disease Clin Orthop Relat Res 1987 225 192 206 3315375

[B20] Whiteside LA Cementless total knee replacement: nine-to 11-year results and 10-year survivorship analysis Clin Orthop Relat Res 1994 309 185 192 7994958

[B21] Lombardi AV Jr Berasi CC Berend KR Evolution of tibial fixation in total knee arthroplasty J Arthroplasty 2007 22 4 Suppl 1 25 29 17570273 10.1016/j.arth.2007.02.006

[B22] Kim T Suzuki M Ohtsuki C Masuda K Tamai H Watanabe E Osaka A Moriya H Enhancement of bone growth in titanium fiber mesh by surface modification with hydrogen peroxide solution containing tantalum chloride J Biomed Mater Res (B Appl Biomater) 2003 64 19 26 10.1002/jbm.b.10469 12474243

[B23] Conditt MA Noble PC Bertolusso R Woody J Parsley BS The PCL significantly affects the functional outcome of total knee arthroplasty J Arthroplasty 2004 19 7 107 112 10.1016/j.arth.2004.06.006 15457428

[B24] Schai PA Scott RD Thornhill TS Total knee arthroplasty with posterior cruciate retention in patients with rheumatoid arthritis Clin Orthop Relat Res 1999 367 96 106 10546603

[B25] Rodriguez JA Saddler S Edelman S Ranawat CS Long-term results of total knee arthroplasty in class 3 and 4 rheumatoid arthritis J Arthroplasty 1996 11 2 141 145 10.1016/S0883-5403(05)80007-5 8648306

